# Efficient and Symmetric Temperature Control in Capillary Electrophoresis II: Thermal Performance When Cooling Capillaries Are Tied Around Analytical Capillaries

**DOI:** 10.1002/jssc.70240

**Published:** 2025-08-13

**Authors:** Leonel Bortolotto Macedo, Cristian Bonatto, Tarso B. Ledur Kist

**Affiliations:** ^1^ Faculty of Pharmacy Federal University of Rio Grande Do Sul Porto Alegre Brazil; ^2^ Institute of Physics Federal University of Rio Grande Do Sul Porto Alegre Brazil; ^3^ Institute of Chemistry Federal University of Rio Grande Do Sul Porto Alegre Brazil; ^4^ Laboratory of Optical Sensors PEA, University of São Paulo São Paulo Brazil

**Keywords:** capillary tying, heat dissipation, Joule effect, separation efficiency, strong fields

## Abstract

Current commercially available capillary electrophoresis instruments lack centrosymmetric and efficient temperature control along the whole extend of the capillary. Here we studied the characteristics and thermal performance of a cooling system that consists of cooling capillaries (fused silica microtubes) which are tied around the complete extent of the analytical capillaries. Six cooling capillaries with only 75 µm inner diameter (320 µm outer diameter—without polyimide) were tied around the outer surface of 320 µm outer diameter analytical capillaries (also without polyimide). The tying process is detailed in a previous publication (*J Sep Sci*. 2025; 48: e70081. https://doi.org/10.1002/jssc.70081). The application of a pressure gradient of 0.25 bar/cm in the cooling liquid was enough to efficiently remove heat and control temperature. Very strong electric fields could be applied, producing very high and stable electric currents. Fields beyond 3500 Volts/cm were applied in a 50 µm inner diameter capillary filled with 20 mM sodium phosphate buffer solution at pH 7.20 and the coolant set at 25°C. This cooling system outperformed the two most used systems: forced air and recirculating liquid coolant in a tube with a capillary inside. The spatial steady‐state temperature profiles of the systems were simulated by numerically solving the heat equation using a finite element method. The centrosymmetric temperature profile and efficiency of this cooling system was corroborated by these numerical results. An objective parameter indicating cooling asymmetry was introduced and used to quantify the superior performance of this new cooling system.

**Abbreviations**
IW: inner wall
OW: outer wall

## 1 Introduction

Capillaries filled with electrically conductive solutions (called BGEs) are required for electrodriven separations in capillary electrophoresis. The use of diluted BGEs restricts eligible samples to diluted samples only, otherwise severe peak tailing would be observed due to electro dispersion. Therefore, Joule heating (or Ohmic heating) will always be present in these conductive BGEs, creating radial temperature gradients. Temperature profiles are usually parabolic within the BGE, where heat is generated, and are descending logarithms in the surroundings where heat diffuses into the coolant [1]. The parabolic temperature profile in the BGE also creates an almost parabolic electrophoretic mobility profile across the capillary lumen. This in turn causes peak dispersion (Taylor–Aris dispersion) in addition to unavoidable molecular diffusion. Consequently, this severely limits the maximum field strengths that can be applied in the separations. In other words, longer capillaries and consequently longer run times are required to achieve high separation efficiencies (*N*) for a given tube bore and BGE. This is equivalent to saying that the radial temperature gradient imposes an upper limit to the “time efficiencies” (*𝒩*)^1^ (Time efficiency (𝒩) is defined on page 138 and summarized in Table 5.5 of ref. [2]) achievable by a given method within a given separation modality (all modalities are listed in [2, append. A]).

These radial temperature gradients have additional detrimental effects on repeatability, separation efficiencies, (*N*) and time efficiency (*𝒩*) that are worth mentioning: i) In some cases they create radial pH gradients in the BGE when temperature sensitive buffers are used. This is common when amines are the conjugated weak acid or weak base in the buffer solution. Although these pH gradients can be used to promote on‐line band compression in some cases [3, 4, 5], in most cases they are disadvantageous. ii) Temperature affects the values of “critical micelle concentration” in micellar electrokinetic chromatography and the values of “affinity constants” in affinity electrophoresis. In most cases these gradients are also disadvantageous. iii) Many measurements must be performed at a defined temperature, but these radial temperature gradients turn everything more complicated and usually require some mathematical corrections.

The above‐mentioned temperature related phenomena are intrinsic to electrodriven separation techniques and occur *inside* the capillary bores, i.e., in the BGE. In addition to this, currently available commercial instruments have three important *outside* imperfections related to temperature control that affect the overall performance of instruments and their ease of use. These imperfections are:

- There are non‐thermostated segments of the separation capillary. Approximately 8 cm of the inlet end and approximately 8 cm of the outlet end are non‐thermostated. This means that there are also temperature differences along the axial direction, and as the non‐thermostated segment of the inlet participates in the separation process itself repeatability and reproducibility are compromised [6, 7, 8].
- There are large temperature differences between what is observed in the “BGE/capillary inner wall (IW)” interface and what is set in the circulating coolant (air or liquid). These temperature differences do not, in themselves, affect separation efficiencies and time efficiencies [2]. However, they do compromise method reproducibility from instrument to instrument and have other undesired effects.
- Temperature control around the external surface of the capillaries is usually not centrosymmetric, as air hits one side of the capillary and produces a turbulent flow pattern on the opposite side ([2, fig. 7.3]). Note that local temperature affects the BGE's local viscosity, which in turn affects its local electroosmotic mobility. These EOF inhomogeneities create local recirculating micro flows that contribute to band dispersion [9, 10].

The transient and steady state temperature radial gradients observed in isotachophoresis conducted in circular cross‐section columns were studied by Coxon and Binder back in 1974 [11]. In the 1990s many important studies were carried out regarding the overall effects of temperature in CE [12, 13], on peak migration times [14], separation efficiency [15], and the influence of the cooling system used on separation efficiency [16]. A detailed study of the effects of axial temperature profiles in CE was conducted by Gaš [17]. More recently, the effect of axial variations of temperature in CE [18] and a procedure to determine temperatures in both efficiently‐cooled part (middle) and inefficiently‐cooled parts (inlet and outlet ends) of the capillary was demonstrated [19].

In the present work a series of prototypes to overcome the three drawbacks mentioned above were produced and tested. To do this, the prototypes should provide efficient and centrosymmetric temperature control along the complete extent of the separation medium. Prototypes were made by tying cooling capillaries around the outer surface of analytical capillaries. The bundles produced were always inspected under a microscope to check for any imperfection. Tying set‐ups that produce good closure using three or more cooling capillaries were addressed in a previous article [20] and some examples are shown by Figure S1. In addition, a video showing the operation of the automatic tying machine that the previous work developed was made available [21]. The thermal performance of this system was compared to that of the most commonly used systems, which are forced air and recirculating liquid coolant in a tube with a capillary inside. Furthermore, the spatial temperature profiles of the systems were calculated by numerically solving the thermal diffusion differential equation using finite elements.

## 2 Material and Methods

### 2.1 Materials

The high voltage (HV) source was a Bertan model 205B (Hicksville, NY) and a voltage divider model V1G from EMCO (Sutter Creek, CA) is used to check the voltage output displayed by the HV source. A digital anemometer (model GM816 from Benetech (Montgomery, IL) was used to measure the wind speed in the force air cooling system. The reagents Na_2_HPO_4_ and NaH_2_PO_4_ were purchased from Sigma/Merck (St. Louis, MO). Filters with 0.22 µm pore size are from Millipore/Merck. Platin electrodes with a diameter of 0.5 mm were also purchased from Sigma/Merck. All capillaries (fused silica microtubes) were obtained from Polymicro/Molex (Phoenix, AZ). The same distilled water with a conductivity of 2.0 µScm^−1^ was used throughout this work, both to prepare the buffer solution (BGE) and as the liquid coolant in the cooling capillaries.

All measurements were taken using one of the most used BGE in CE: a 20 mM sodium phosphate buffer solution at pH 7.20. It was prepared by mixing 36 mL of 0.2 M Na_2_HPO_4_ with 14 mL of 0.2 M Na_2_H_2_PO_4_, then diluting this mixture to 500 mL in a volumetric flask using distilled water. The conductivity of this solution was 2.68 mScm^−1^ at 25°C. The pH of phosphate buffer solutions is insensitive to temperature around neutral pH, i.e., the p*k*_a2_ of phosphate exhibits dp*k*_a2_/dT ∼ 0 around neutral pH [4].

**FIGURE 1:**
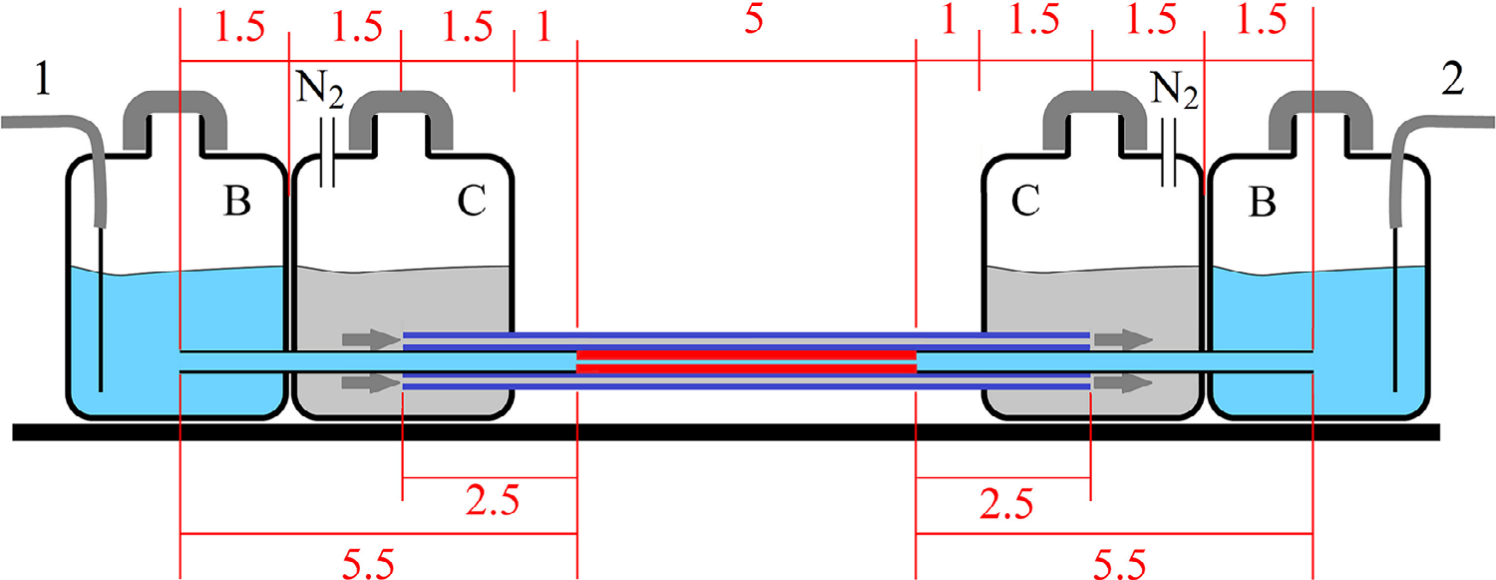
A vertical cut of the cooling system proposed in the present work. Only two cooling capillaries (dark blue) can be seen in this vertical cut. The analytical capillary (red) is 5 cm long and the extension capillaries (with 320 µm ID and 430 µm OD) are 5.5 cm long on each side (black). The six cooling capillaries are tied around the analytical capillary as shown in ref. [20, 21]. The BGE (light blue) located in the BGE reservoirs (B) fills the analytical capillary (red) as well as the reservoir's extensions (black). The coolant (light gray) is pressure driven using nitrogen gas through the cooling capillaries, from the left coolant container (C) to the right, or the opposite. The BGE reservoirs (B) containing the electrodes (1 and 2) are electrically and hydrodynamically isolated from the coolant containers (C).

The electric currents versus applied voltages and respective thermal performances (stability and repeatability) were measured using cooling capillaries tied around the analytical capillaries as depicted in Figure 1. The cooling capillaries start and finish inside the coolant containing reservoirs. Nitrogen gas from a cylinder with a pressure regulator was used to pressure drive the coolant from any one of the two coolant reservoirs to the other one. The cooling capillaries used were 10 cm long, therefore extending 2.5 cm beyond the inlet and 2.5 beyond the outlet of the analytical capillaries and assuring whole extension temperature control. The tips of the analytical capillaries were fused (using fusion splicing, as detailed in ref. [22]) to 5.5 cm segments of wide bore capillaries (320 µm ID and 430 µm OD). Unlike connections made using glue, these all‐fused silica compact connections withstand alkali solutions, strong acids, high temperatures, and extremely high electric field strengths. They exhibit good thermal conductivity, mechanical stability, and compatibility with the surrounding cooling capillaries. The 5.5 cm end segments work as extensions of the reservoirs, and the heat generated in these wide bore segments is small compared to the 5 cm analytical capillaries with a much thinner ID. The observed voltage drop along these 5.5 cm segments (or reservoir extensions) is very small compared to that in the 5 cm analytical capillary, which means that the applied HV *U* (0 Volts at one electrode and 0 up to 30 kV at the opposite electrode) drops almost entirely along the 5 cm of the analytical capillary. The effective axial electric field strength experienced by the BGE inside the analytical capillary will be, in good approximation, given by *E* = 0.95 U/5 cm (50 µm ID and 5 cm long analytical capillary and two segments of 320 µm ID and 5.5 long reservoir extension) and *E* = 0.89 U/5 cm (75 µm ID and 5 cm long analytical capillary and two segments of 320 µm ID and 5.5 long reservoir extension), where *U* is the HV difference applied between the electrodes (1 and 2 in Figure 1). The factors 0.95 and 0.89 were obtained assuming a constant conductivity in all segments. The electric currents observed during the operation of the systems were measured by placing a 55.6 kΩ and 50 W resistor in series with the grounding wire and by measuring the electric potential difference over this resistor. Sample injection, separation, detection, repeatability, separation efficiencies (*N*), and time efficiencies (*𝒩*) [2] will the subject of forthcoming articles.

The same distilled water, filtered with a 0.22 µm pore size filter and with a conductivity of 2.0 µScm^−1^, was used as the coolant in all experiments. We observed that distilled water outperforms most dielectric liquids in almost all properties that are important for closed cooling systems (thermal conductivity, specific heat capacity, viscosity, flammability, toxicity, freezing point, boiling point, chemical stability, cost, availability, and green credentials). This is in good agreement with the theory; see, for instance, [16, tab. IV]. There is one important exception: if the analytical capillary breaks it releases the BGE into the coolant water, which makes it conductive. Fortunately, there are easy and safe ways to prevent any exposure to the HV if this happens: i) the reservoirs of the coolants are made of glass or polymer and are kept electrically isolated from everything else. ii) From time‐to‐time the conductivity of the cooling water must be measured to detect any contamination.

## 3 Results and Discussions

### 3.1 Experimental Results

There are many possible set‐ups of cooling capillaries tied around analytical capillaries. Here are a few of them: i) analytical capillaries with polyimide or without polyimide (two possibilities); ii) cooling capillaries with or without polyimide (two possibilities); iii) analytical capillaries with 150 µm OD (120 µm without polyimide) or 360 µm OD (320 µm without polyimide) (two possibilities); iv) analytical capillaries with 50 or 75 µm ID (can be more) (two possibilities); v) using three, four, five, six, seven, or eight (can be more) cooling capillaries (six possibilities); vi) cooling capillaries with small ID, medium, or large IDs (three possibilities); vii) coolant flowing only through the lumen of the cooling capillaries (when glue is used in the tying process [20, 21]), coolant only flowing along the triangular voids (tied without glue), or coolant flowing along both (three possibilities). Combining these set‐ups alone gives 2 × 2 × 2 × 2 × 6 × 3 × 3 = 864 possible set‐ups to be tested (in triplicates this would require 2592 prototypes).

**FIGURE 2:**
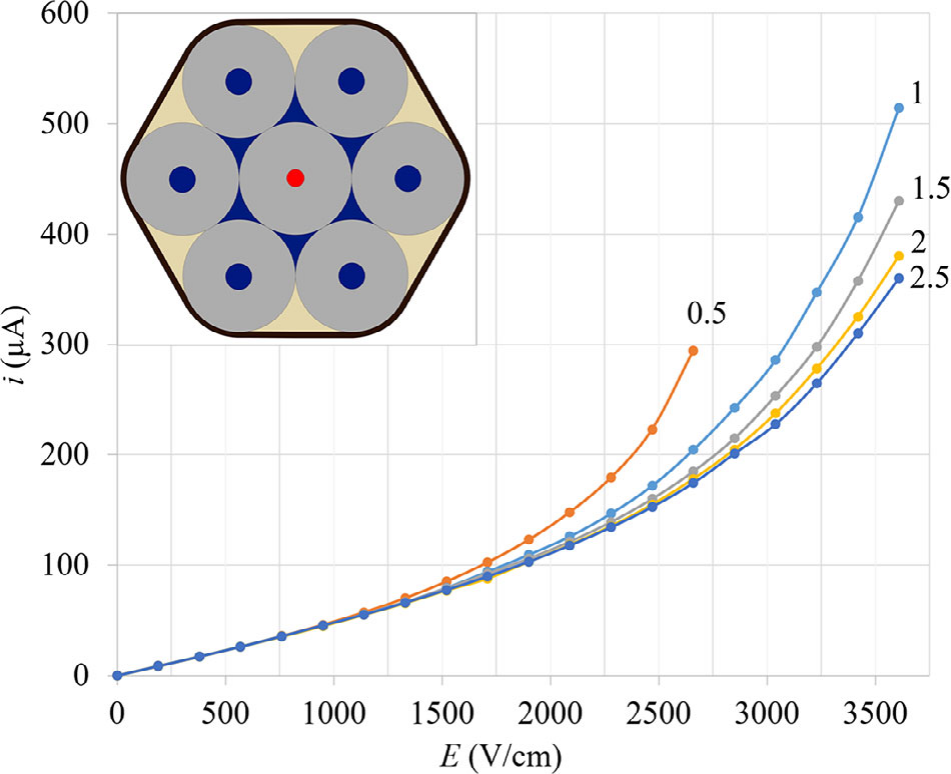
The observed electric currents (*i*) at 25°C along a 5 cm long analytical capillary with 50 µm ID and filled with 20 mM sodium phosphate buffer solution at pH 7.20. They were measured as a function of the effective applied electric field (*E*, experienced by the BGE within the analytical capillary) and the applied nitrogen pressure on the coolant reservoir (shown in Figure 1). The inset shows a cross section of the bundle of capillaries. The coolant (blue) flows through the lumen of the six cooling capillaries (75 µm ID and 320 µm OD) and through the voids between the analytical and cooling capillaries. The epoxy glue (yellow) was used only externally after the tying process. The tying thread is shown in black (for details see [20, 21]).

**FIGURE 3:**
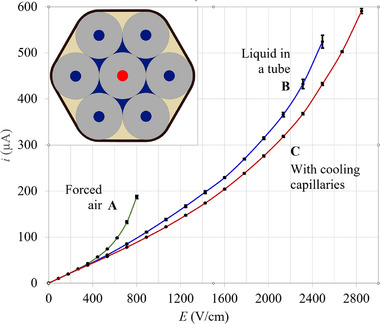
This graph compares the observed electric currents in an analytical capillary with 75 µm ID and 320 µm OD as a function of the electric field strength, with the coolant set at 25°C, for three distinct cooling systems: forced air (A), recirculating liquid coolant (B), and with cooling capillaries tied around the analytical capillaries (C). Note the high and stable electric currents in C. The inset shows the cross section of the bundle of capillaries with the analytical capillary (BGE is in red) surrounded by six cooling capillaries (75 µm ID and 320 µm OD).

In the present work six cooling capillaries without polyimide were tied around the analytical capillary (also without polyimide) (shown in the inserts of Figures 1 and 2). No glue was used during the tying process, only externally to the bundle, after the tying process. The analytical capillaries (with 50, 75, or 100 µm ID) have 320 µm OD and all cooling capillaries have only 75 µm ID and 320 µm OD (prior to polyimide removal their OD was 360 µm). The coolant (distilled water) flows along the lumen of the six cooling capillaries (blue disks of the insets of Figures 2 and 3) as well as along the voids between the analytical capillary and the cooling capillaries (blue triangles with curved sides or circular arcs). Note that this cooling system meets all the three missing properties discussed in the introduction:

- The whole extent of the analytical capillary is thermostated (shown in Figure 1).
- It is efficient, as the coolant flows close to the heat source using a structure that is entirely made of quartz (which has one of the highest dielectric breakdown voltages among all known materials). Its cooling efficiency is proven in Sections 3.2 to 3.5.
- It is a centrosymmetric cooling system. The coolant flows through the six triangular voids and through the lumens of the six cooling capillaries. This symmetry is demonstrated in Section 4.

### 3.2 The Required Pressure Gradient

The pressure gradient required to efficiently control temperature with such thin cooling capillaries (75 µm ID and 320 µm OD) was checked. For comparison, the cross‐sectional area of the lumen of each cooling capillary is (4417 µm^2^) and the cross‐sectional area of each triangular void is (4128 µm^2^). A series of measurements (three prototypes and three measurements of each prototype) of the electric currents running through the analytical capillary (50 µm ID), taken as a function of the applied electric field strengths, were made by applying 0.5, 1, 1.5, 2, and 2.5 bar of nitrogen gas to drive the coolant (distilled water) through the lumen of the 10 cm long cooling capillaries and through the voids between the cooling capillaries and the analytical capillary (see inset of Figure 2). This was done to discover the minimum pressure required to efficiently thermostate the analytical capillary. The results show (Figure 2) that applying 2.5 bar for 10 cm long cooling capillaries provides satisfactory cooling. This gives the coolant a pressure gradient of 0.25 bar/cm. Note the extremely strong electric field applied in this case.

The data of Figure 2 shows that the application of 2.5 bar is enough to stabilize the *i* × *E* relationship, making it asymptotically reach the lowest values. Therefore, the pressure gradient experienced by the coolant (distilled water) is 0.25 bar/cm. Similar results were obtained for 75 and 100 µm ID analytical capillaries. Note the extremely high field strengths and electric currents observed.

### 3.3 Comparison With Existing Cooling Systems

It is important to compare the most used cooling systems with the one proposed in the present work. Prototypes were produced for both the forced air and recirculating liquid coolant system. Some measures were taken to make this caparison fair:

- The same 5.5 cm long (Figure 1) wide bore end extensions were used in all prototypes, including forced air cooling and recirculating liquid cooling. Therefore, the whole analytical capillary (5 cm long) was exposed to the flux of air (at 2.2 m/s) and to the recirculating liquid coolant (700 mL/min delivered from a thermostated bath).
- Polyimide was removed from both analytical capillary and reservoir extension. This is to facilitate thermal flow in these systems.
- For the recirculating liquid cooling system, the 5 cm long analytical capillary and the 5.5 cm extensions were placed right at the center of a 4 mm ID, 6 mm OD, and 10.5 cm long quartz tube. The coolant entrance (quartz “T” connector) is in the middle of the 5.5 cm inlet extension and the exit “T” in the middle of the 5.5 cm outlet extension, maximizing cooling efficiency of the analytical capillary in this way (drawings of these set‐ups are shown in Figures S2 and S3).

Note that in commercial instruments approximately 8 cm of capillary at both ends is not thermostated at all. To complicate matters even more, most of the thermostated section of the capillary usually sticks to the IW of the coolant‐containing polymer tubing, caused by electrostatic attraction. This compromises the efficiency of the cooling process in commercial instruments.

As shown in Figure 3, the close to ideal recirculating liquid cooling system (blue curve) works much better than the forced air system (green curve). However, the set‐up of cooling capillaries tied around the analytical capillaries performed better (red curve) than the recirculating cooling system with a flow rate of 700 mL/min of distilled water. It also sustains much higher electric field strengths. The average temperature of the BGE, for a given electric field strength, is lower in C than to B. The standard deviations (vertical bars) are from nine measurements (from three prototypes and three measurements on each prototype). Note that the standard deviations for the cooling system proposed here (red curve) is much lower than the others (A and B). This is indicative of a more reproducible (three prototypes) and stable operation, without temperature fluctuations caused by turbulent airflow (in A) or liquid (in B).

### 3.4 Numerical Results of Temperature Profiles

The steady‐state thermal profile was simulated by solving the heat equation using the finite element method implemented in the open‐source FEniCS platform [23]. The heat equation, (∂^2^/∂*x* ^2^ + ∂^2^/∂*y* ^2^) *T* = *p*/*λ*, was solved using linear Lagrange elements (where *p* is the heat generated per unit volume in the BGE and *λ* is the heat transfer coefficient). In the tied‐capillaries configuration, a 1000 µm radial domain with a resolution of 300 elements along the characteristic radius was used. For the lateral cooling configuration, a rectangular 1000 µm × 1000 µm domain with 600 elements per direction was employed. In both cases, three‐level adaptive refinement was applied in the heat source region, along with automatic refinement at material interfaces. Additional refinement near the boundaries was introduced in the lateral cooling configuration to better resolve the imposed temperature conditions. A heat source of 76 W/m was applied only in the BGE in all simulations.

The following heat transfer coefficients were used throughout this work: 0.6 Wm^−1^K^−1^ for water (blue part of inset of Figure 2) and BGE (red in Figure 3), 1.4 Wm^−1^K^−1^ for quartz (gray in Figure 2), and 0.2 Wm^−1^K^−1^ for epoxy glue (yellow in Figure 2). A coolant fixed at 25°C was used as the boundary condition.

**FIGURE 4:**
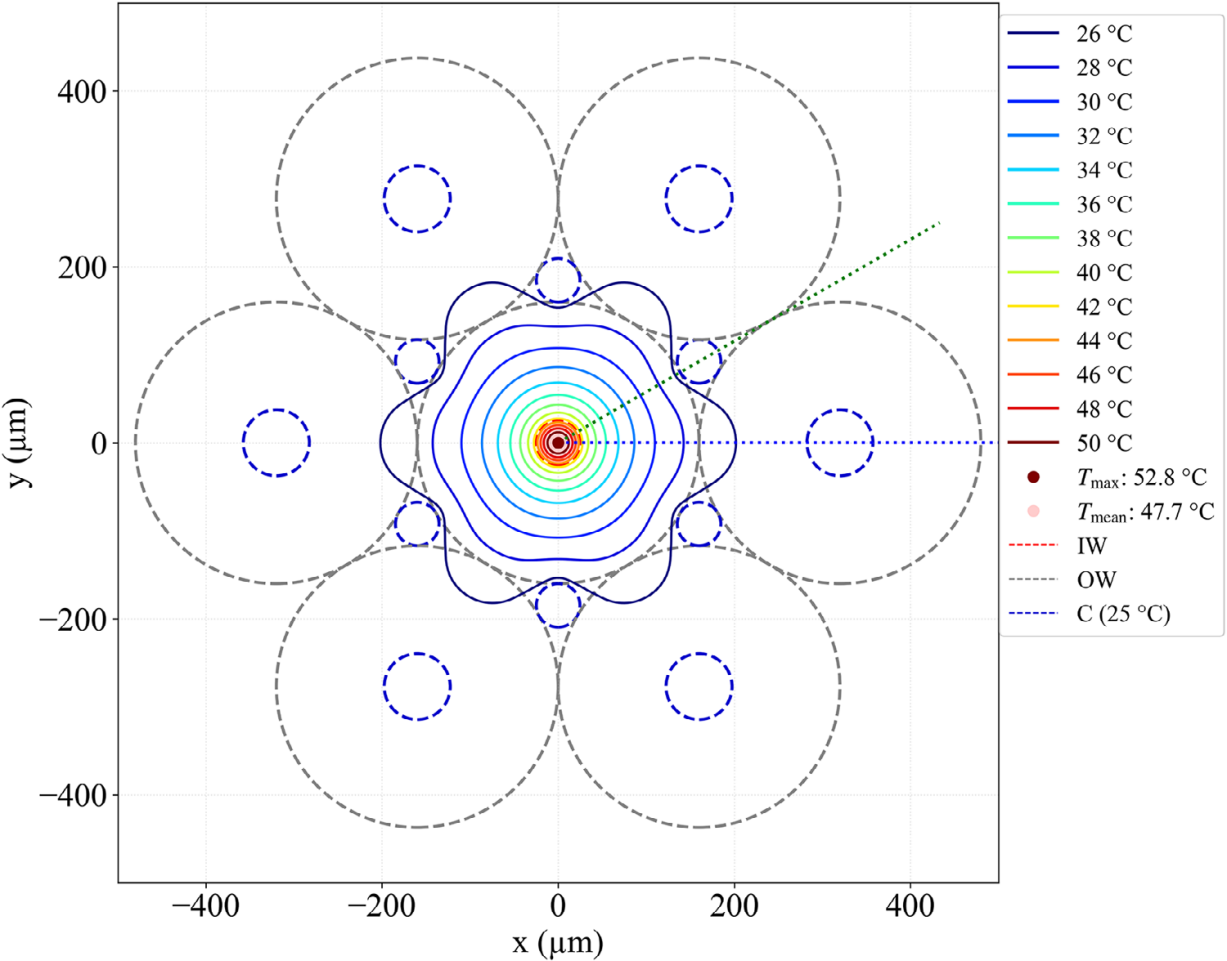
Isotherms of the system shown in the inset of Figure 2 (50 µm ID), operated with the coolant at 25°C and the BGE subjected to 76 W/m. The coolant flows along the lumen of the cooling capillaries (75 µm ID and 320 µm OD) as well as along the triangular voids between the cooling capillaries and analytical capillary, both without polyimide. For the triangular voids, the approximation that the temperature is constant (25°C) across the cylinders that insert into the voids (six inner blue dashed circles) was used. IW stands for inner wall (BGE/fused silica interface), OW for fused silica capillaries outer wall, and C for coolant. *T*_max_ is the maximum temperature in the BGE and *T*_mean_ is the mean temperature in the BGE. Note the centrosymmetric geometry of the isotherms in the BGE phase.

**FIGURE 5:**
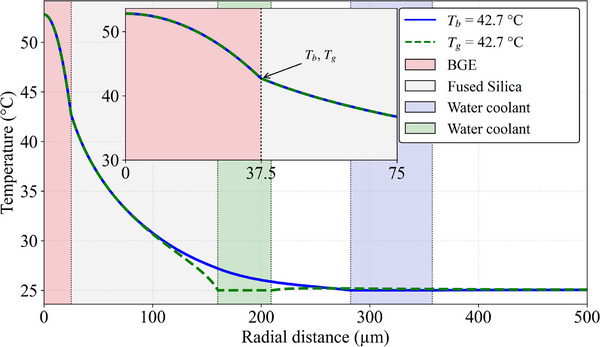
The radial temperature profiles of the system shown in inset of Figure 2 (ID = 50 µm). One profile passes through the center of a cooling capillary (blue line) and the second runs along the contact point of two neighboring cooling capillaries (green line). These lines are shown in Figure 4. The inset at the center top shows the details of the temperature profile at the inner wall interface. *T*_b_ is the temperature at the IW along the blue line while *T*_g_ is the temperature at the IW along the green line. The difference between *T*_b_ and *T*_g_ is less than 0.05°C. Both curves have a parabolic form from *r* = 0 to *r* = 25 µm and a decreasing logarithm from 25 to at least ∼50 µm. Note that temperature exhibits an almost perfect parabolic profile inside the BGE.

Figure 4 shows the isotherms of the set‐up shown in the inset of Figure 2. In the simulations the coolant flowed at 25°C through the six lumens of the cooling capillaries (six outer blue dashed circles) and through the six triangle‐like cross sections. The flow efficiency is low at the corners of these triangle‐like cross sections. Therefore, in the simulations the temperature was kept at 25°C inside the inscribed cylinders (six small blue dashed circles). The central red dashed circle shows the position of the capillary IW in Figure 4. It is worth mentioning the highly centrosymmetric shape of these isothermal surfaces in the BGE region, even at such high power of heat generated per unit volume, which led to *T*_max_ = 52.8°C at the capillary centerline. The isothermal contours change from circular in the BGE to corrugated at the outer wall (OW) (see the isothermals in blue at 28°C and darker blue at 26°C in Figure 4). This symmetry is reached at the midpoint between the capillary IW and capillary OW (see the lighter blue isothermal at 32°C). Note that *T*_max_ − *T*_mean_ = 5.1°C, consequently the maximum temperature difference within the BGE in this case is Δ*T*_in_ = 2 × 5.1°C = 10.2°C, because the mean height of a paraboloid is half its maximum^2^. Another way to calculate this is using Δ*T*_in_ = *T*_max_ − T_mean,IW_, where *T*_mean,IW_ is the average temperature at the capillary IW (this can be found in Figure 5). Δ*T*_out_ is the temperature difference observed outside the BGE and this is given by Δ*T*_out_ = *T*_mean,IW_–25°C = 47.7°C – 5.1°C–25°C = 17.6°C. All of this was calculated assuming constant electrical conductivity in the BGE. In practical situations, an increase in temperature also increases the local conductivity, which in turn increases the local heat generated per unit volume compared to volume elements that are at a lower temperature.

Figure 5 shows two temperature profiles along the radial direction of Figure 4, one passing through the center of a cooling capillary (blue line) and the second along the contact point of two neighboring cooling capillaries (green). This shows the centrosymmetric profile of the isotherms. The maximum temperature difference between these two profiles at the capillary IW (*T*_b_ − *T*_g_) is less than 0.05°C per 10.2°C of Δ*T*_in_ (temperature difference within the BGE). Additional isotherms for capillaries with 75 µm and 100 µm ID are given in the Figures S4 to S7. The isotherms of capillaries with 3–8 cooling capillaries also given in the Figure S8 to S13).

Finally, we used the same software code to find the temperature isotherms of the commonly used forced air system. In our model the air hits one side of the capillary and with a turbulent flow on the opposite side. The following parameters were used in this study: air density (or specific mass) of *ρ*_m_ = 1.1839 kg/m^3^ (at 25°C), air velocity of *v* = 10 m/s, capillary outer diameter of 360 µm OD, and air dynamic viscosity of *η* = 18.46 × 10^−6^ Pa.s (at 25°C). The Reynolds equation is given by *R* = *ρ*_m_ *v* OD/(2 *η*). This produces a Reynolds number of *R* ∼ 115. The expected airflow patterns for many ranges of *R* are given in [2], fig. 7.3 and Figure S14. The simulations were made using the coolant (air) set at 25°C and with the same heat rate used in the previous simulations, 76 W/m.

**FIGURE 6:**
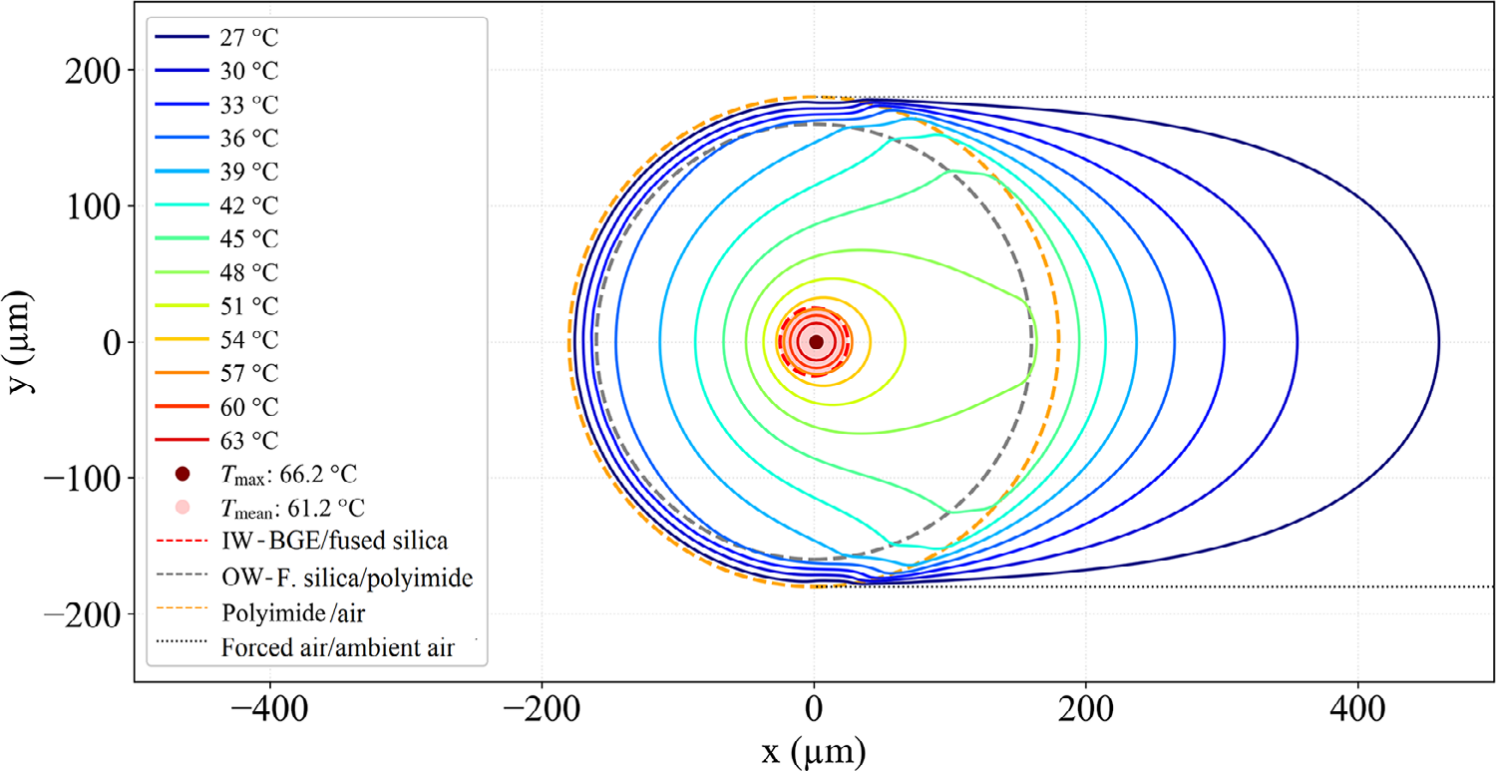
Steady state isotherms of a full developed system when air at 25°C hits one side of the capillary. The turbulent pattern on the opposite side is not included in the simulations. The capillary has 75 µm ID and is operated at 76 W/m. Polyimide is 20 µm thick (a layer between the gray dashed circle and the yellow dashed circle) and the capillary total OD is 360 µm.

Figure 6 shows the spatial temperature profile of a fully developed system. A severe asymmetry can be observed in the spatial temperature profile. This cooling situation is still good for the conventional system. In real practical situations, many capillary segments are stuck on the IW of the cartridge or even stuck in a corner. In these segments, the air flows at a lower velocity and only over one side of the capillary, rather than over half of the circumference. This has the potential to produce even greater asymmetries in the temperature profiles and higher temperatures in the BGE. Not forgetting that ∼8 cm of the capillary both end segments of current commercially available instruments are not thermostated at all [6, 7]. This produces severe limitations to CE as an analytical technique.

**FIGURE 7:**
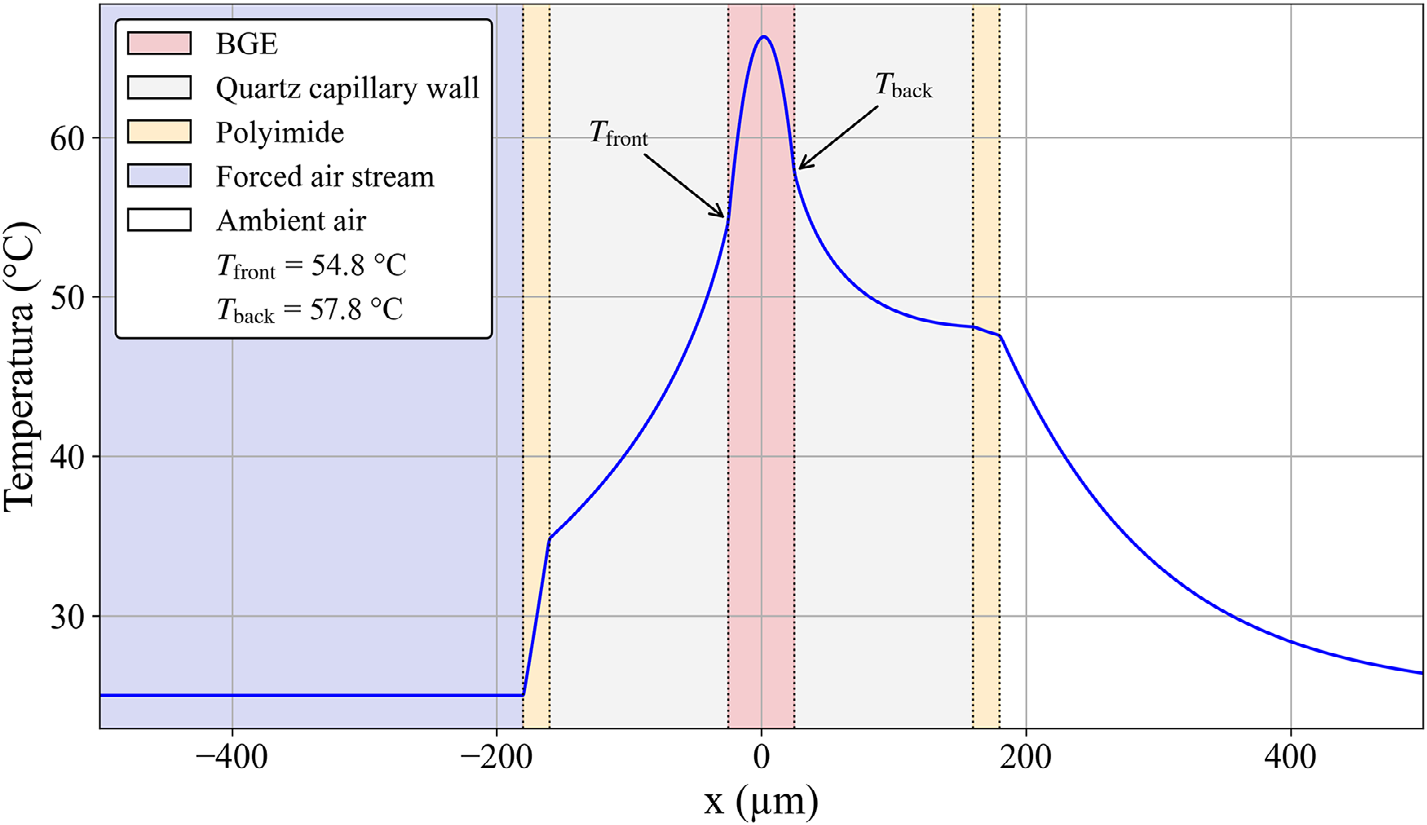
Full developed and steady state temperature profile of the simulation of a commonly used cooling system (forced air). Note the high temperature differences in the capillary inner wall. It is 57.8°C on one side and 54.8°C on the opposite side. Moreover, in this case the temperature profile in the BGE is a decentralized and skewed parabola. For lager IDs this is even more pronounced (see Figures S15 to S18).

The spatial temperature profile of Figure 6 is shown in Figure 7. The capillary IW temperature is ∼50.6°C on one side and ∼55.2°C on the opposite side. This gives a temperature difference of ∼4.6°C, which is much higher than the < 0.05°C observed in Figure 5. Moreover, the maximum temperature is not along the centerline. This creates many disturbances, including in the local electroosmotic flow. As mentioned before, local temperature affects the BGE's local viscosity, which in turn affects its local electroosmotic mobility. These EOF inhomogeneities create local recirculating micro flows that contribute to band dispersion [9, 10].

Table 1 summarizes the temperature profiles obtained from the simulations for analytical capillaries with 50, 75, and 100 µm ID under the following two conditions: using the cooling system presented in this work and a forced air cooling system. The heating power per unit length was kept constant at 76 W/m for all simulations, and the coolant temperature was kept at 25°C for all. Note *T*_max_ and *T*_mean_ are significantly higher in the forced air cooling system than in the system proposed in this work. As a result, the temperature set on the cartridge in commercial instruments often does not reflect the actual temperature experienced in the BGE, and may be misleading.

**TABLE 1:** A summary of the temperatures given by the simulations and the proposed parameter indicating cooling asymmetry (*α*).

		Cooling system					
	Units, symbols, or calculation mode	Cooling capillaries tied around the analytical capillaries			Forced air hitting one side of the analytical capillary		
Capillary ID	µm	50^a^	75	100	50^a^	75	100
Maximum *T* in the BGE	$T_{\max}$	52.8	49.3	46.8	66.2	62.8	60.4
Mean *T* in the BGE	$T_{\mathrm{mean}}$	47.7	44.3	41.8	61.2	57.8	55.4
Maximum *T* at capillary inner wall	$T_{\max,\mathrm{IW}}$	42.7	39.3	36.7	57.8	55.2	53.7
Minimum *T* at capillary inner wall	$T_{\min,\mathrm{IW}}$	42.7	39.3	36.7	54.8	50.6	47.5
Mean *T* at capillary inner wall ($T_{\mathrm{mean},\;\mathrm{IW}})$	$T_{\mathrm{mean},\mathrm{IW}}≃\frac{T_{\max,\mathrm{IW}}+T_{\min,\mathrm{IW}}}{2}$	42.7	39.3	36.7	56.3	52.9	50.6
Δ*T* inside the BGE ($\Delta T_{\mathrm{in}}$)^b^	$\;\Delta T_{\mathrm{in}}≃T_{\max}-T_{\mathrm{mean},\mathrm{IW}}$	10.1	10.1	10.1	9.9	9.9	9.8
Δ*T* outside the BGE ($\Delta T_{\mathrm{out}}$)	$\;\Delta T_{\mathrm{out}}≃T_{\mathrm{mean},\mathrm{IW}}-25$	17.7	14.2	11.7	31.3	27.9	25.6
Cooling asymmetry factor (*α*)	$\alpha =\frac{T_{\max,\mathrm{IW}}\;-\;T_{\min,\mathrm{IW}}}{\Delta T_{\mathrm{in}}}$	< 0.005	< 0.005	< 0.005	0.30	0.46	0.63

^a^ The isotherms of these are shown in Figures 4 and 5. For the other IDs they are shown in the Supporting Information.

^b^ It can also be calculated using Δ*T*_in_ = 2(*T*_max _‐ *T*_mean_) based on the property shown in the Footnote 2.

The cooling asymmetry factor (*α*) represents the number of degrees Celsius of temperature difference at the capillary IW per degree Celsius of Δ*T*_in_ that is build up and is sustained by the Joule effect. A lower *α* value indicates a more symmetric cooling system.

Δ*T*_out_ tends to be large if the coolant flows far from the heat source (BGE) and/or when low thermal conducting materials, such as polyimide and/or air, are present in between. Therefore, set‐ups in which coolant flows close to the BGE and at high shear rates tend to perform best.

## 4 Conclusion

The use of cooling capillaries with only 75 µm ID tied around the whole extent of analytical capillaries was found to be effective in regulating temperature. Distilled water at 25°C was used as the coolant. Very strong electric fields could be applied, resulting in stable, albeit high, electric currents. The system operated steadily with fields beyond 3500 Volts/cm in a 50 µm ID capillary, filled with 20 mM sodium phosphate buffer solution at pH 7.20 and with the coolant set to 25°C. The standard deviation bars of nine measurements (three prototypes with three measurements taken for each) of this system are much smaller than those of the two most used: forced air and recirculating liquid coolant. An explanation for this may be the high shear rate and absence of turbulence in the coolant fluid of the new proposed cooling system. The simulations corroborated the observed cooling efficiency and showed centrosymmetric temperature isotherms in the BGE. A centrosymmetric cooling system maintains the same temperature along all points of a cross section of the “BGE/capillary IW” interface. If this is performed along the whole extent of the analytical capillary then new possibilities will be within reach. This opens new possibilities in CE. BGE can be carefully designed or “engineered”, using the dp*k*_a_/dT and other properties of the BGE constituents, to mitigate the band dispersion caused by Joule heating (Taylor–Aris dispersion). This has the potential to allow CE to be operated at higher electric currents and under stronger electric fields, delivering higher separation efficiencies (N) and higher time efficiencies (*𝒩*) [2] when using wide bore analytical capillaries (e.g. 100 µm) and/or BGEs with higher conductivities. Due to the high stability of the electric currents, it is expected that the configuration presented in the present work will also present greater repeatability in migration times and quantification. Finally, it is worth highlighting that it may be hard to devise a cooling system that is more efficient, reliable, and compact compared to this one demonstrated in this work.

## Author Contributions

T.B.L.K. conceived the presented idea, developed the prototypes, wrote the manuscript, and provided all materials, reagents, and instruments (except the PC used in the simulations) with his own funding. L.B.M. assembled the prototypes and performed the experimental measurements. C.B. developed and wrote the code to solve the heat equation using the “finite elements” method. After validating the code by comparing the output data with a known problem with analytical solutions, he also performed the simulations. All authors discussed the results and contributed to the final manuscript.

## Conflicts of Interest

The authors declare no conflicts of interest.

## Supporting information




**Supporting File 1**: jssc70240 sup 0001 SuppMat.pdf

## Data Availability

The data that supports the findings of this study is available from the author upon reasonable request.

## Endnotes

1 This character is given by MS Word italic monotype corsiva and LaTex code ∖mathcal{N}.

2 The average height of a paraboloid $(z=1-\frac{r^{2}}{a^{2}})\;$ with 0 ≤ *r* ≤ *a* is given by: $\;\frac{1}{\pi a^{2}}\int_{0}^{2\pi }d\theta \int_{0}^{a}\left(1-\frac{r^{2}}{a^{2}}\right)\;rdr=\frac{1}{2}$.
