# Supplementary material for: Efficient and Symmetric Temperature Control in Capillary Electrophoresis II: Thermal Performance When Cooling Capillaries Are Tied Around Analytical Capillaries
Source: J Sep Sci. 2025 Aug 13;48(8):e70240. doi: 10.1002/jssc.70240 (PMC12351157; doi:10.1002/jssc.70240)
Supplement: Supplementary file 1 — Supporting File 1: jssc70240 sup 0001 SuppMat.pdf [file JSSC-48-e70240-s001.pdf]

SUPPLEMENTARY INFORMATION

Efficient and Symmetric Temperature Control in Capillary Electrophoresis II: Thermal Performance when Cooling Capillaries are Tied Around Analytical Capillaries.

Leonel Bortolotto Macedo[1], Cristian Bonatto[2], and Tarso B. Ledur Kist[3,4,]

[1]Faculty of Pharmacy, Federal University of Rio Grande do Sul, Porto Alegre, RS, Brazil
[2]Institute of Physics, Federal University of Rio Grande do Sul, Porto Alegre, RS, Brazil
[3]Institute of Chemistry, Federal University of Rio Grande do Sul, Porto Alegre, RS, Brazil
[4]Laboratory of Optical Sensors, PEA, University of São Paulo, São Paulo, SP, Brazil

Correspondence: Tarso B. Ledur Kist (tarso.kist@gmail.com)
Phone: + 55 51 3308 7175. Fax: +55 51 3308 7304

1 | Introduction

This SI text contains the following supplementary information divided in the following sections:

2. Tying Options of Commercially Available Capillaries
3. The prototypes of 'forced air' and 'recirculating liquid coolant' used in this work
4. Additional isotherms of cooling capillaries tied around the analytical capillaries
5. The air flow pattern around analytical capillaries cooled with forced air
6. Additional isotherms of the forced air cooling system

2 | Options of Commercially Available Capillaries

It can be challenging to find commercially available cooling capillary models that are a good fit around the analytical capillary, i.e., without piling up or leaving a void between the first and last cooling capillary. Figure 1 shows a drawing, to scale, of some possibilities using commercially available models of analytical capillaries and cooling capillaries (one analytical capillary surrounded by three, four, five, six, seven, and eight cooling capillaries). In the present work the set-up of n = 6 was chosen, but without polyimide, which gives and outer diameter (OD) of 320 μm instead of 360 μm.

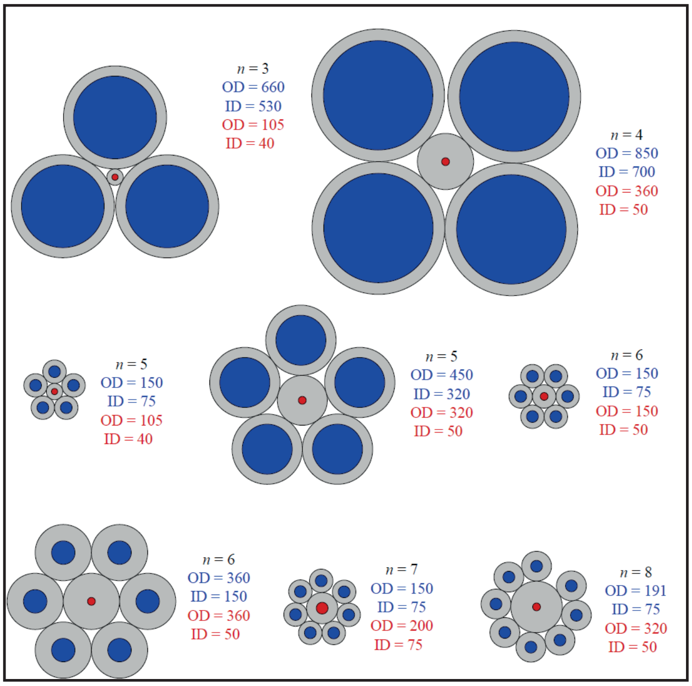

FIGURE 1 | Drawing (to scale) showing the best combinations of analytical capillaries (central) and n cooling capillaries (n = 3 to 8), considering commercially available capillary models. Blue numbers refer to the cooling capillaries and red numbers to the analytical capillaries. Polyimide coatings are not shown in these figures. Note that there are still some voids left (on the upper right side of some sets). In the present work we chose option n = 6, but without polyimide (OD = 320 μm).

3 | The prototypes of 'forced air' and 'recirculating liquid coolant' used in this work

The commonly used cooling systems (forced air and recirculating liquid coolant in a tube) where compared with the one studied in the present work (cooling capillaries tied around the analytical capillary). Prototypes were carefully prepared (all in triplicate) to make this comparison as fair as possible (see Fig. 2 and 3). Note that in all systems the whole extent of the analytical capillary is exposed to the air flow (in the forced air system of Fig. 2) and to the recirculating liquid coolant (liquid in a tube shown in Fig. 3). Moreover, in all prototypes (depicted in Figs. 2 and 3) the capillaries are without polyimide.

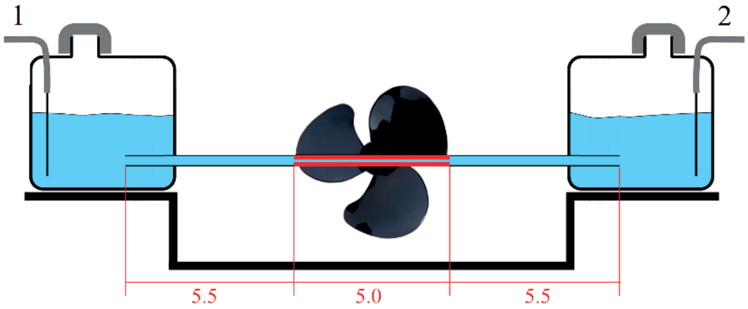

FIGURE 2 | A schematic drawing of the forced air cooling system with the numbers in cm. The analytical capillary (red) is 5 cm long and without polyimide (with ID 50, 75 or 100 µm and 320 µm OD). The reservoir extensions (with 320 µm ID and 430 µm OD) are 5.5 cm long on each side (black) of the analytical capillary. The BGE (light blue) located in the BGE reservoirs fills the analytical capillary (red) as well as the reservoir's extensions (black). The electrodes 1 and 2 are indicated and an electric potential difference U is applied between them. Note that the whole analytical capillary is exposed to the air flow blown by the fan.

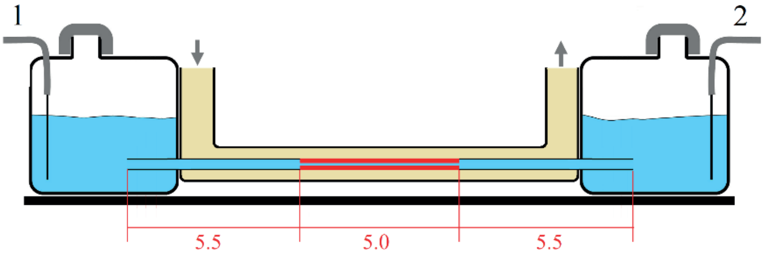

FIGURE 3 | A schematic drawing of the recirculating liquid cooling system. The same as in Fig. 2 but with a recirculating liquid instead of air flow. Note that the whole analytical capillary (without polyimide) is exposed to the liquid coolant (light yellow) that flows continuously from a thermal bath through the "U" shaped quartz tube with 6 mm OD and 4 mm ID.

4 | Additional isotherms of cooling capillaries tied around the analytical capillaries

Figure 4 shows the isotherms of the simulations of an analytical capillary with 75 µm ID. Note that the same amount of 'heat per unit length' (76 W/m) is used in all cases, i.e., power per unit length is constant in all simulations and not power per unit volume. T_{max} is lower here (49.3 °C) than it is observed of a 50 µm ID capillary (52.8 °C), and ΔT_{in} = 10.0 °C and ΔT_{out} = 14.3 °C. All of this happens because part of the heat is generated closer to the coolant.

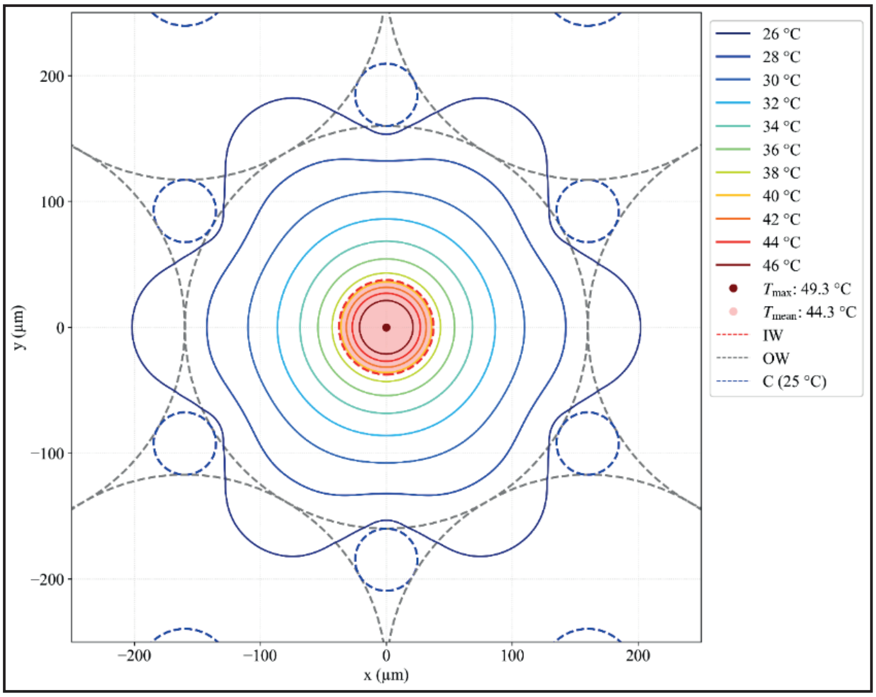

FIGURE 4 | Isotherms of the system when am analytical capillary with 75 µm ID is used and operated with the coolant at 25 °C. The applied power is the same as in all cases (76 W/m).

Figure 5 shows two radial temperature profiles of Fig. 4, one passing through the center of a cooling capillary (blue line) and the second passing along the contact point of two neighboring cooling capillaries (green). Note the centrosymmetric shape of these profiles in the BGE and deep in the analytical capillary wall. Only close to the surface do they start to diverge. The difference between T_b and T_g in this case is smaller than 0.05 °C.

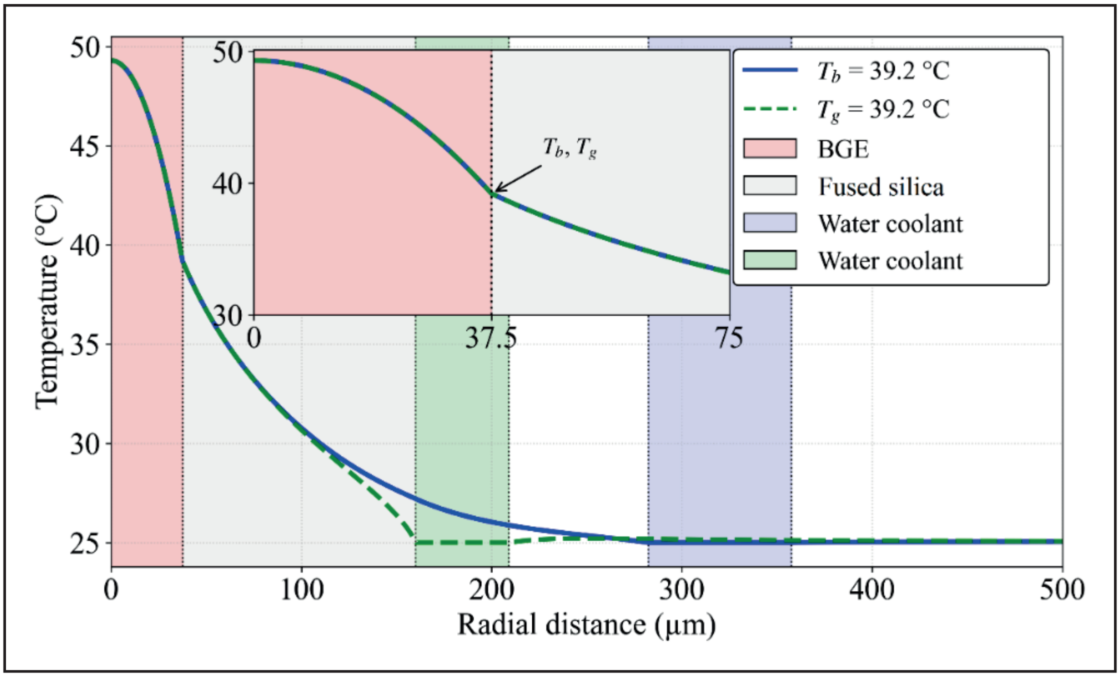

FIGURE 5 | The radial temperature profiles of Fig. 4 (75 µm ID). The inset at the center top shows the details of the temperature profile at the inner wall (IW) interface, it is T_b along the blue line and T_g along the green line. T_b is the temperature at the IW along the blue line while T_g is the temperature at the IW along the green line (blue and green lines are shown in Fig. 4 of the main text). The difference between T_b and T_g is less than 0.05 °C. Both curves have a parabolic form from r = 0 to r = 25 µm and a decreasing logarithm from 25 to at least ~50 µm.

Figure 6 shows the isotherms when a 100 μm ID capillary is used. In this case T_{max} (46.8 ºC), T_{mean} (41.8 ºC), $\Delta T_{max,IW}$ (36.7 ºC), $\Delta T_{max,IW}$ (36.7 ºC), and ΔT_{in} (1.1 ºC) are all smaller than in the previous cases (with 50 and 75 μm ID). Nonetheless, a good centrosymmetric temperature profile can still be seen.

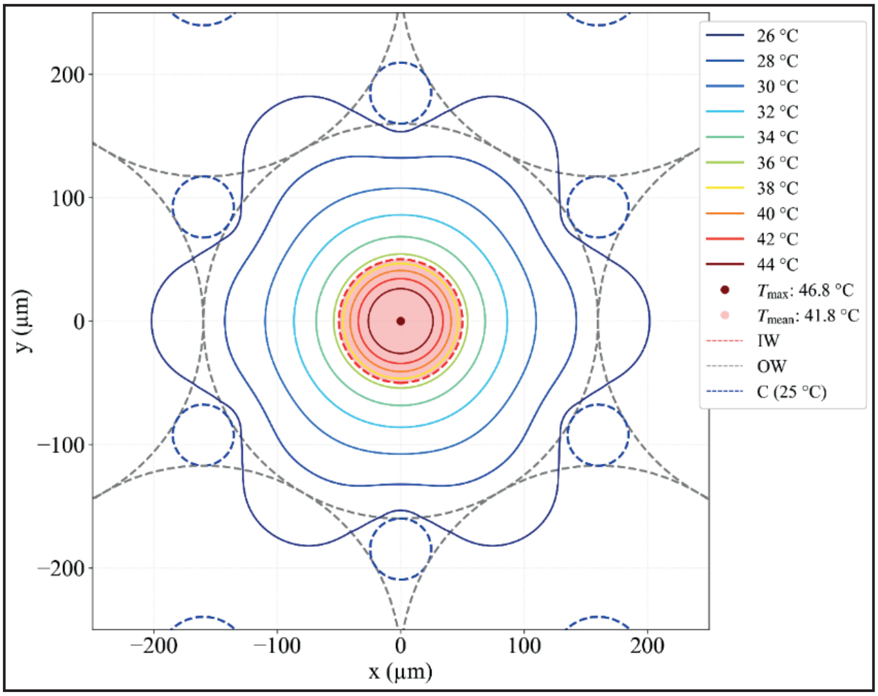

FIGURE 6 | Isotherms of the system for a capillary with 100 um ID and with the coolant at 25 ºC. Note that Tmax is even lower in this case. The applied power (76 W/m) used was the same for all simulations.

Figure 7 shows the radial temperature profiles of Fig. 6. The temperature profiles are still centrosymmetric within the BGE. This occurs due to the large number of flow paths of the coolant (6 + 6). In this case the difference between T_b and T_g is also smaller than 0.05 ºC.

The use of such wide bore capillaries is highly desired, as it improves LOD and LOQ. However, they are rarely used in practice because of the severe peak dispersion (Taylor-Aris dispersion) observed when they are operated at the same electric field strengths as used with the 50 and 75 μm ID capillaries.

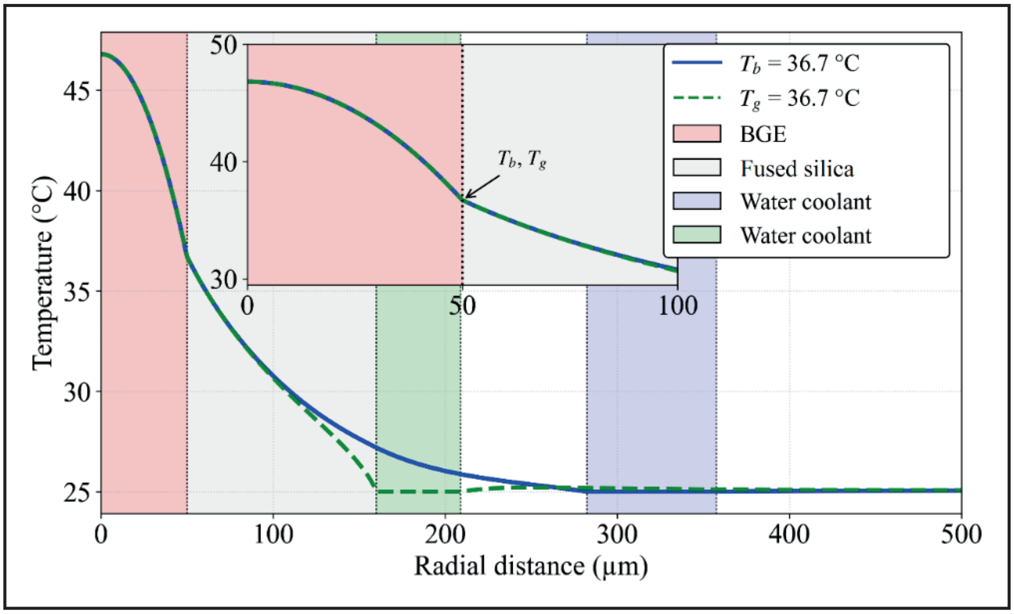

FIGURE 7 | The radial temperature profiles of Fig. 6 (100 μm ID) and same notation as of Fig. 5.

Figures 8 to 13 show the isotherms when using n = 3 to 8 cooling capillaries (as shown in Fig. 1). In all cases the coolant was set at T = 25 °C and the same power per unit length was applied (76 W/m). However, unlike in the previous cases, the triangular void between the analytical capillary and the cooling capillaries is filled with epoxy glue (with a thermal conductivity of 0.2 W·m^{-1}·K^{-1}) in all the cases shown in Figs. 8 to 13. Moreover, commercially available capillaries (shown in Fig. 1) typically have a polyimide coating. To simplify the simulations, we considered the capillaries to be made entirely of quartz.

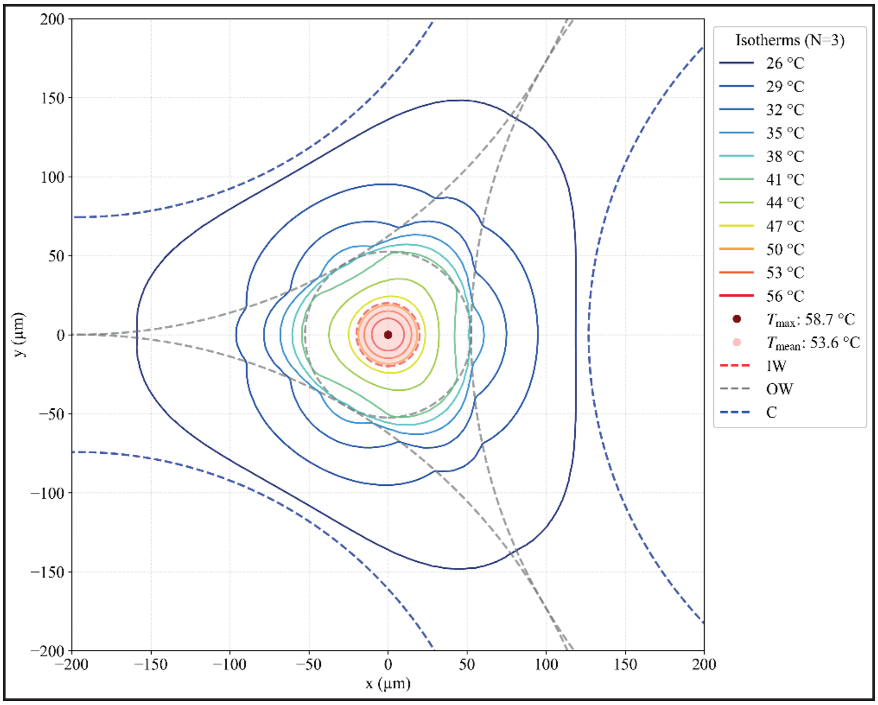

FIGURE 8 | Isotherms when three ($n = 3$) cooling capillaries are used. The dashed gray arcs are the outer surface of the cooling capillaries and the dashed blue lines are the inner surface of the cooling capillaries (shown in Fig, 1). The blue dashed line is set at T = 25 °C in the simulations.

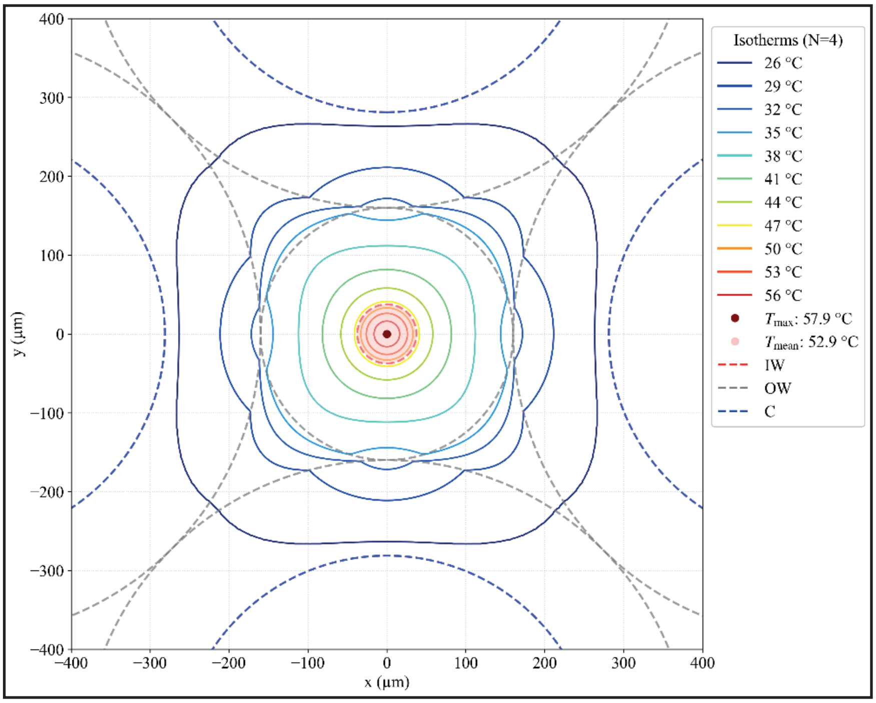

FIGURE 9 | Isotherms for $n = 4$ and analog to the drawing shown in Fig 1, but without the void at the top. The other conditions are the same as in Fig. 8.

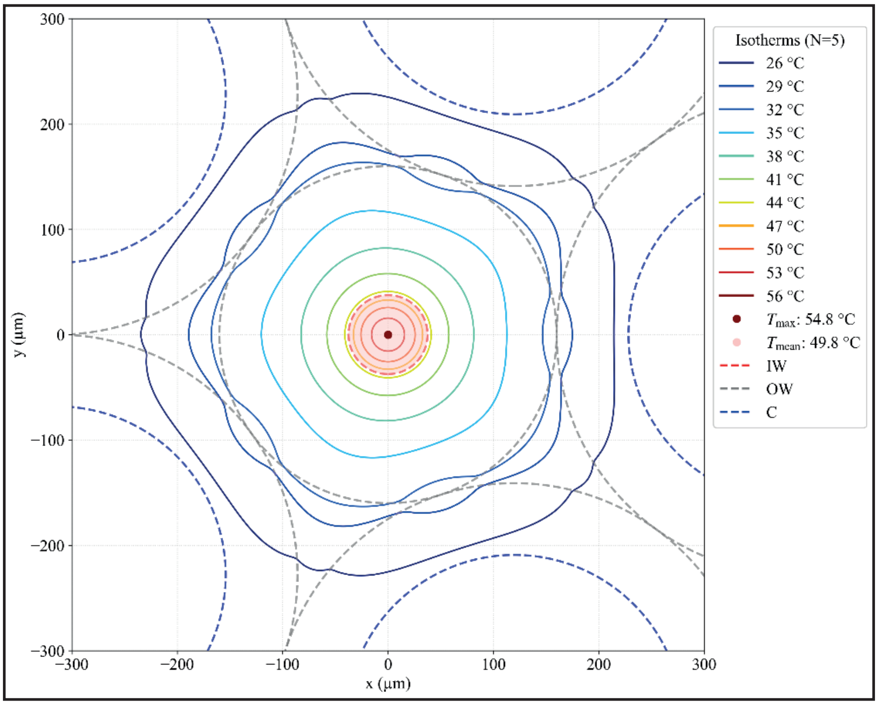

FIGURE 10 | Isotherms for n = 5 and analog to the drawing shown in Fig 1. The other conditions are the same as in Fig. 8.

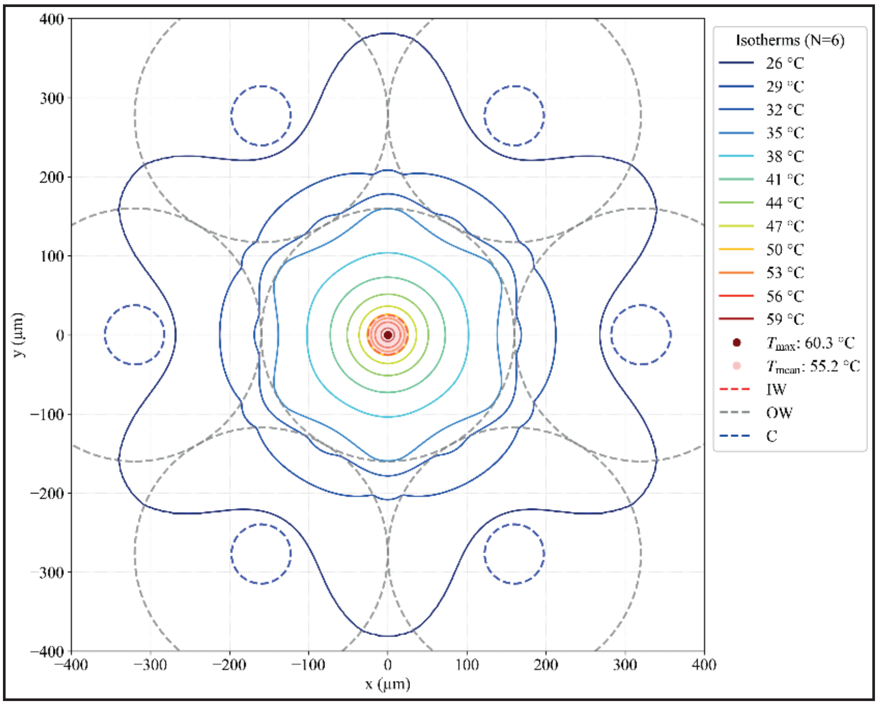

FIGURE 11 | Isotherms for n = 5 and analog to the drawing shown in Fig 1. The other conditions are the same as in Fig. 8.

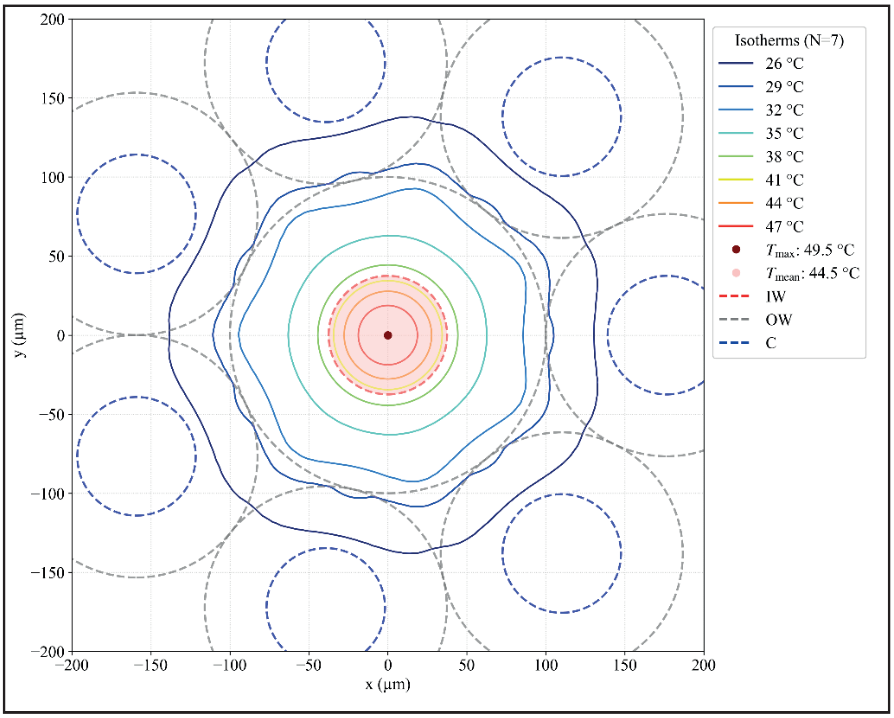

FIGURE 12 | Isotherms for n = 7 and analog to the drawing shown in Fig 1, but without the void at the upper right side. The other conditions are the same as in Fig. 8.

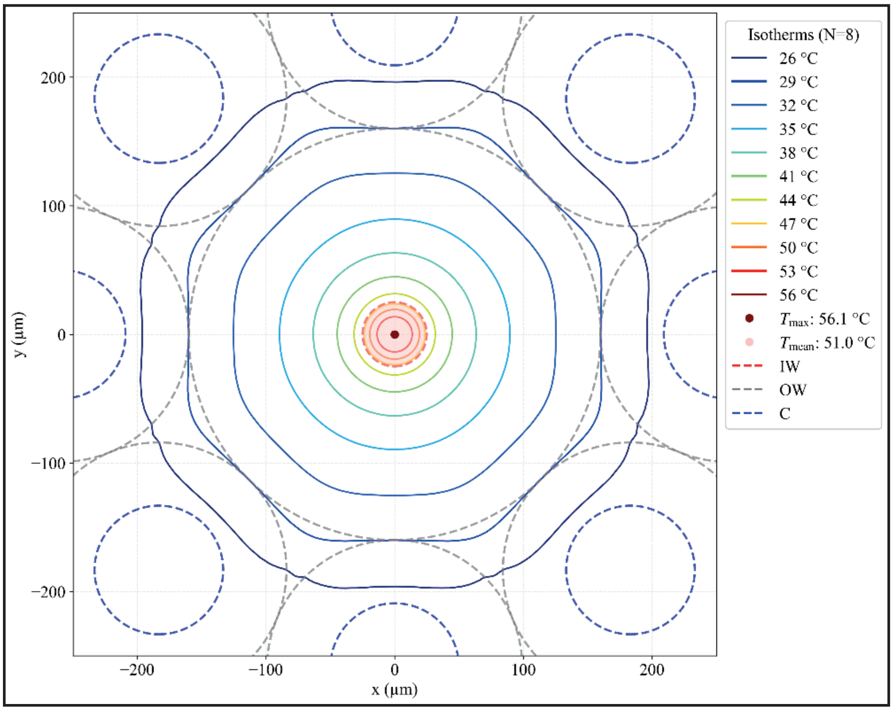

FIGURE 13 | Isotherms for n = 8 and analog to the drawing shown in Fig 1, but without the void at the upper right side. The other conditions are the same as in Fig. 8.

5 | The air flow pattern around analytical capillaries cooled with forced air

Figure 14 A shows the airflow pattern of the most used cooling system (forced air). The Reynolds number of a typical condition is R ≈ 115 (calculated in the main text). Therefore, the expected airflow pattern is given by Fig. 14 A (see Fig. 7.3 of [1]). The simulations were made using the coolant (air) set at 25 °C and with the border conditions shown in Fig. 14 B. Heat was generated in the BGE in the lumen of the capillary (75 μm ID) at the same rate used in the previous simulations, 76 W/m. The complex turbulent pattern of the right side of Fig. 14 A was not considered in the simulations. In practice the temperature of the air increases as it contours the capillary outer surface from left to the right. In the simulations this temperature was considered constant (25 °C), as shown in Fig. 14 B (blue).

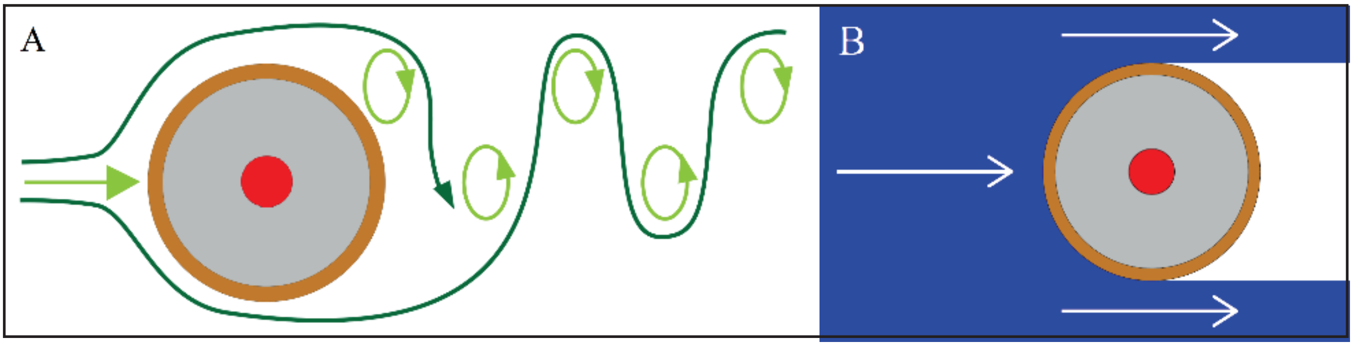

FIGURE 14 | The expected airflow pattern around a capillary segment that is totally exposed to the air flow when a forced air-cooling system is used in CE (A). The simulations were made using a 360 μm OD capillary, which includes the 20 μm layer of polyimide (brown). In this drawing the capillary has 75 μm ID (red) and is operated with the same power (P = 76 W/m) of the previous simulations. The coolant (air) was set at 25 °C (blue) and hits one side of the capillary outer surface with a speed of 10 m/s (B). Figure 14 A was taken from [1] with permission.

6 | Additional isotherms of the forced air cooling system

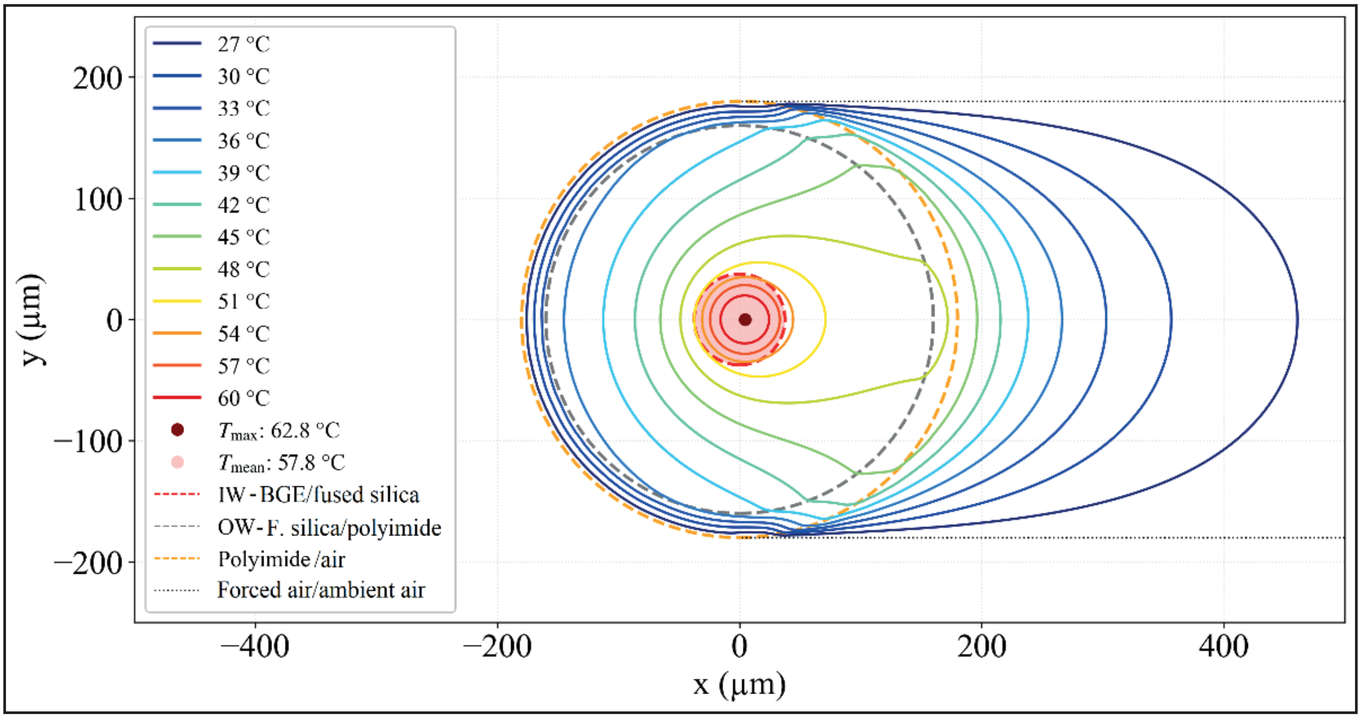

FIGURE 15 | Steady state isotherms of a full developed system when air at 25 °C hits one side of the capillary. The turbulent pattern on the opposite side is not included in the simulations. The capillary has 75 μm ID and is operated at 76 W/m. Polyimide is 20 μm thick (a layer between the gray dashed circle and the yellow dashed circle) and the capillary total OD is 360 μm.

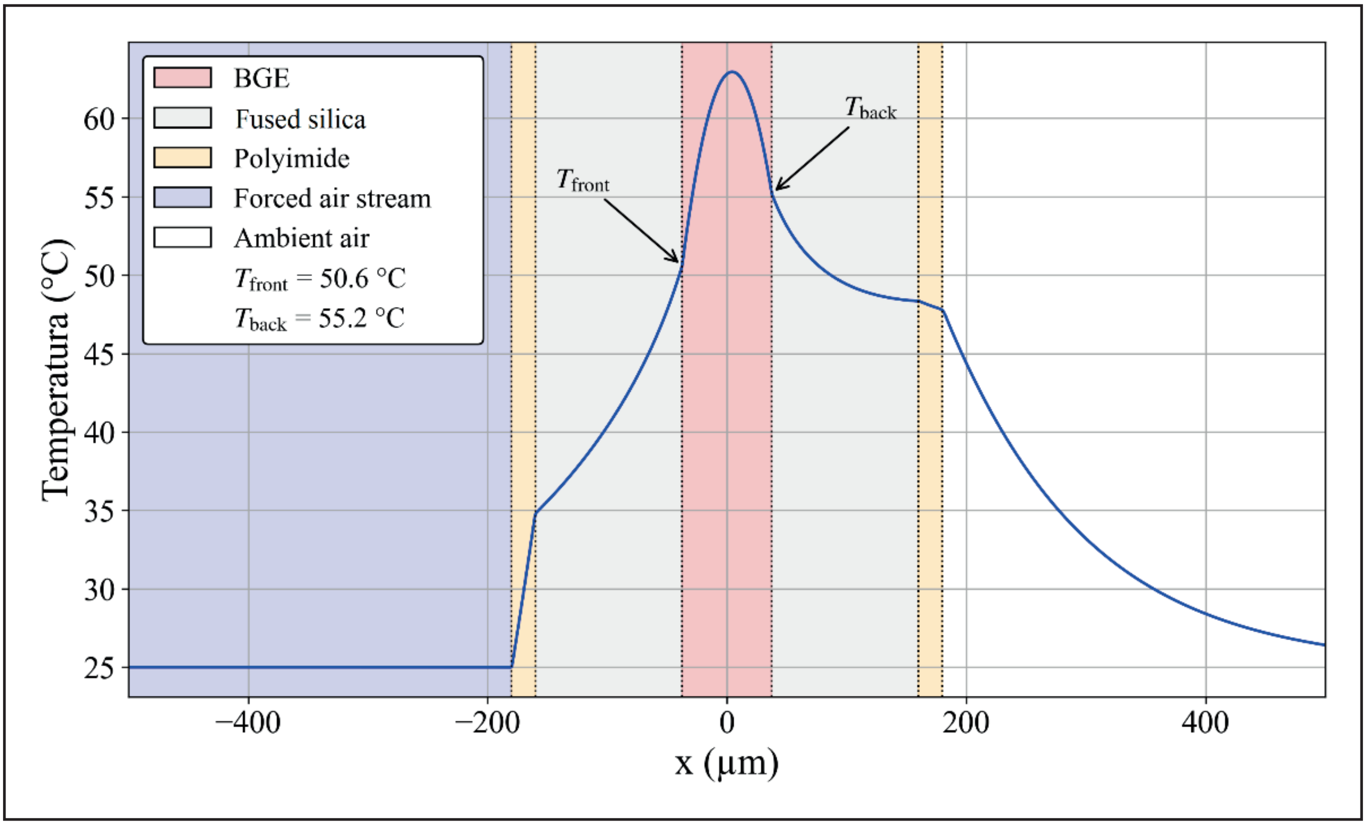

FIGURE 16 | Full developed and steady state temperature profile along the air-flow direction of Fig. 15 (forced air). Note the high temperature differences in the capillary inner wall. It is 55.2 °C on one side and 50.6 °C on the opposite side.

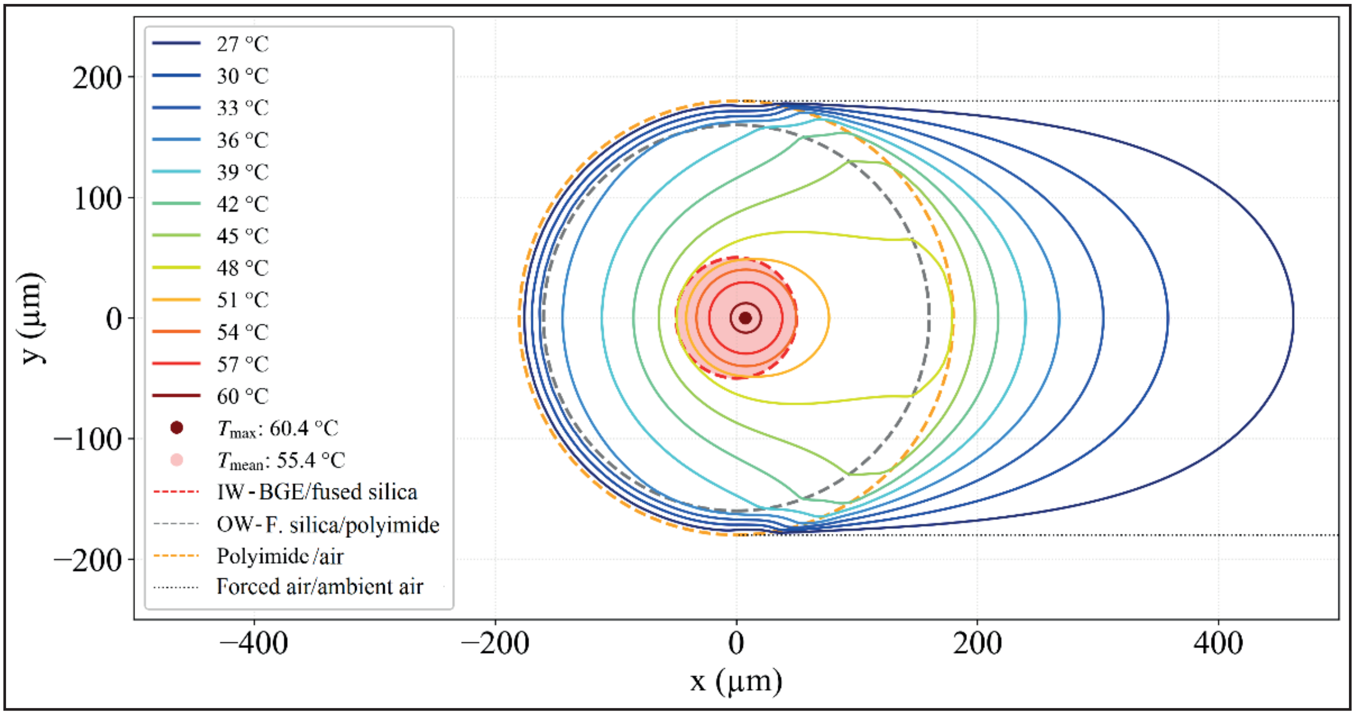

FIGURE 17 | Temperature isotherms of a capillary with 100 µm ID. The other conditions are the same as in Fig. 15.

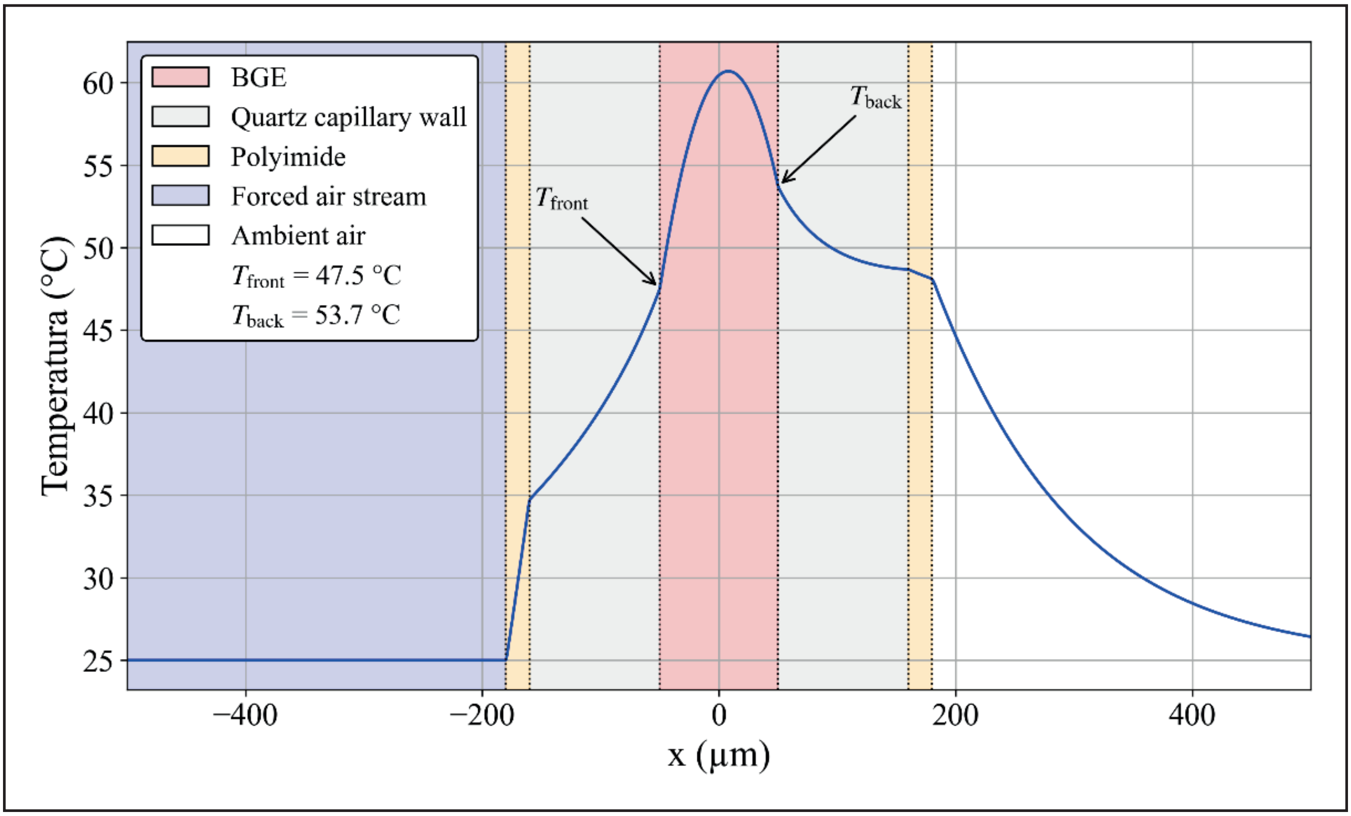

FIGURE 18 | Temperature profile along the air-flow direction of Fig. 17 (forced air) and along the center of a capillary with 100 μm ID. Note that the high temperature differences in the capillary inner wall are even higher in this case, 53.7 °C on one side and 47.5 °C on the opposite side.

REFERENCE

[1] Kist TBL. Open and Toroidal Electrophoresis, 1st Ed. Chichester: Wiley; 2020.
